# Changes in Selected Cognitive and Motor Skills as Well as the Quality of Life After a 24-Week Multidimensional Music-Based Exercise Program in People With Dementia

**DOI:** 10.1177/15333175231191022

**Published:** 2023-08-23

**Authors:** Alexander Prinz, Anneke Schumacher, Kerstin Witte

**Affiliations:** 1 210449 Otto-von-Guericke-University, Magdeburg, German

**Keywords:** dementia, cognition, motor performance, multidimensional music-based exercise program, significance statement

## Abstract

The loss of independence is one of the greatest consequences of dementia. Multidimensional music-based exercise programs could counteract. The present study investigates the effects of such a program on people with dementia and bases on a 24-week intervention with three measurement time points. Sixty-nine people with dementia were randomly assigned to the intervention (n = 43) and control group (n = 26). The following outcome parameters were measured: leg strength, gait, grip strength, balance, reaction time, selected cognitive abilities, and quality of life. A mixed ANOVA with repeated measurement showed significant interaction effects between group and time. After 24-weeks in contrast to the control group the intervention group significantly improved in leg strength (*P* = .001), balance (*P* = .001), gait (*P* = .001), grip strength (right *P* = .002, left *P* = .011), reaction time (*P* = .003), global cognition (*P* = .039), verbal fluency (*P* = .002), attention (*P* = .013) and quality of life (*P* = .011). In conclusion, the program enhanced selected cognitive and motor skills and quality of life.

A music-based exercise program can be helpful for people with dementia to stabilize or improve selected motor and cognitive skills. Non-pharmacological treatments are necessary and should be incorporated into successful dementia management. In addition, music-based exercise programs can be a promising adjunct to conventional drug therapies for people with dementia. The study confirms the results of several studies showing the positive effects of music-based exercise programs.

## Introduction

As a result of demographic change, preventing age-related diseases and avoiding the general decline in performance is becoming increasingly important. Among the most common diseases, besides cardiovascular diseases and diabetes, is dementia. Due to society‘s higher life expectancy, the WHO predicts that dementia patients will triple by 2050.^1^ As dementia leads to a loss of independence, an increased need for long-term care can be expected.^2^ About half of the people with dementia currently require personal care. The other half will develop such a need over time.

Furthermore, about half of the older people in need of care and four-fifths of older people in nursing homes have dementia.^2^ These numbers will increase due to demographic change. Consequently, the global cost of dementia is also expected to rise. In 2019, the costs amounted to 1.3 trillion US$.^1^ By 2050, the cost of care is expected to rise to nearly US$2.8 trillion, showing that this development can become a social problem.^1^ Opportunities must be created to stabilize or improve cognitive and motor skills to counteract this development. As a result, older people would maintain independence and a high degree of need for care would be prevented for as long as possible.

At the moment, this prevention is preferably attempted with drugs. However, the drugs developed against dementia have limited efficacy and are primarily approved for Alzheimer’s disease.^1^ They are also often accompanied by side effects.^3^ In addition, several other drug projects had to be discontinued after unsuccessful trials with patients. For example, Cummings et al^4^ showed that tested dementia drugs had a 99.6% failure rate in clinical trials from 2002 to 2012. The potential drugs failed due to lack of efficacy, excessive side effects or problems with study conduct.^4^ Interest in dementia drug research has decreased after disappointing clinical trials.^1^ Therefore, non-pharmacological interventions are of high importance.^3^

Some studies already show that various non-pharmacological interventions positively affect motor and cognitive skills and quality of life in people with dementia.^5^ Based on these findings, the German Society of Psychiatry and Psychotherapy, Psychosomatics, and Neurology’s S3 guideline on dementia emphasize the importance of non-pharmacological interventions and recommend their implementation.^6^ The content of this evidence-based guideline includes statements on prevention, diagnosis, and therapy of dementia. The aim is to provide those dealing with dementia patients a systematically developed decision-making aid for diagnostics, therapy, and care.^6^ Exercise, as well as music programs, could show particularly positive effects in this regard. For example, music has the highest evidence level in the S3 guidelines on dementia^6^ because music has three main characteristics in dementia therapy. First, it is easily accessible and applicable and has no side effects.^7^ Second, the musical memory are preserved into the late stages of the disease.^8^Among other things, this includes learning new songs^9^ and showing emotional responses to music, such as joy.^10^ Finally, music can also support other (non-musical) functions.^7^ For example, music can stimulate autobiographical memory,^11^ encourage learning, practice, and cognitive training to intensify social interactions and prevent social isolation.^7^ Likewise, music motivates people with dementia, making them participate in programs for a longer time and thus move and exercise more.^12,13^

Furthermore, it can engage brain regions involved in neural scaffolding through its broad network of capabilities.^7^ In addition, music increases the willingness to participate in sports activities, especially in people with dementia.^14^ However, this only happens if it is perceived as pleasant and familiar.^15^

In addition to music programs, exercise programs have also been shown to produce positive effects in people with dementia.^6^ In this context, multidimensional exercise programs, which include components of strengthening, cognition, coordination and balance, affect cognitive and motor skills and the quality of life in people with dementia.^16-18^ These programs had positive effects on walking speed, balance, muscle strength, visual processing, and cognitive abilities, improving everyday functioning and enabling independent living.^17,18^Based on these findings, some studies investigated the combination of music and exercise.

It has been demonstrated that the propensity to adapt movements to rhythm naturally evolved at a very early age and is probably hardwired in humans, as shown by cognitive and neuroscience.^19^ Therefore, the compelling link between music and movement has been harnessed to enhance individual performance and improve health and well-being.^19^ For example, one study has shown that music-based exercise can reduce pain perception during exercise so that the exercise can be performed longer.^20^ Likewise, a positive effect of music-based exercise programs on motivation and participation was found.^21^ The combination of music and exercise also showed positive effects in older persons. Cognitive^22^ and motor skills^23^ and physiological characteristics^24^ could be positively influenced. Despite these positive effects, the combination of music and exercise in dementia patients has been little researched.^25^ Unfortunately, the data of these few studies are heterogeneous, and the results do not have sufficient evidence.^3,26^ However, they give first insights that combining music and movement can have promising effects on motor and cognitive skills.^27,28^ For example, Gomaa et al^25^ show increasing evidence that music-guided exercise can improve some motor and non-motor impairments associated with dementia, including mobility, cognition, and participation. However, it should be noted that the music is often used as passive music in the background or that the exercise program focuses on only one component (strengthening or coordination).^25^ Active use of music to increase training and intensity by performing to the beat of the music or multidimensionality of the exercise program has also been little studied and could be more effective.^26,29^ For example, it was shown that an active and interactive type of music intervention, music with movement, is the most recommended for people with moderate dementia.^30^ This is because expressive and relational skills can be better developed, thus promoting new learning strategies and improving well-being.^31^ Nevertheless, more research is needed in this regard.^3,25,26^

Therefore, it is essential to conduct further robust studies on non-pharmacological treatments focusing on music-based exercise programs, as these could be essential for effective dementia management.^25,32^

Therefore, this pilot-study aims to develop a music-based multidimensional exercise program and investigate its impact on selected cognitive and motor skills and the quality of life.

The primary outcome was to examine the time course (time and time*group) of an intervention group that completed a music-based multidimensional exercise program compared to a control group with no further intervention on selected motor and cognitive skills. The secondary outcome was to examine the time course (time and time*group) of the intervention group compared with a control group on quality of life. Following the above literature, we hypothesized that an intervention group receiving a music-based multidimensional exercise program with the content of a multidimensional intervention would develop better motor and cognitive performance (Hypothesis 1) and quality of life (Hypothesis 2) than an inactive control group.

## Methods

### Study Design

The present pilot-study was designed as a 24-week intervention study with two groups (intervention (IG) and control group (CG)) and three measurement time points (Baseline- (T0), intermediate- (T1)- and Post-test (T2)). The groups were formed by block randomization with unequal group sizes.^33^ The intervention was divided into two 12-week periods, separated by the intermediate test. The research protocol conformed to the principles of the Declaration of Helsinki and was approved by the Ethics Committee of the Otto von Guericke University Magdeburg (Germany) (registration number: 100/20). Data collection took place in December 2020 (Baseline (T0)), March 2021 (Intermediate (T1)), and August 2021 (post-test (T2)) at the respective facilities. The intervention was conducted from January 2021 to July 2021. Due to the Corona pandemic, the intervention was suspended in the homes for approximately two to three weeks. Written informed consent was obtained from participants’ legal representatives in advance. In addition, participants were informed in detail about the purpose of the study at the first meeting.

### Sample Description

Potential subjects were searched for in Magdeburg from October to November 2020. For this purpose, various facilities for people who have dementia were contacted. A total of ten care facilities were contacted. Four care facilities responded and participated. The nursing staff recruited subjects from the facilities in Magdeburg because they knew the potential participants better. For this purpose, the relatives or legal representatives of the potential subjects were contacted and informed about the study in consultation with the cooperating institutions. Their consent was obtained by an informed consent form. However, some legal representatives did not want their relatives to participate in an exercise program. The nursing staff was aware of this problem and was therefore able to make a preliminary selection. The following inclusion criteria guided the nursing staff: Participants needed to be older than 70 years, have mild to moderate dementia (raised with the Mini-Mental-State Examination (MMSE)), and be able to follow an exercise program and walk around on their own or with a walker. Exclusion criteria were: Hypertension, severe cardiovascular diseases such as cardiac arrhythmias, renal insufficiency, and severe motor impairment.

Power analysis with G*Power 3 (version 3.1.9.7, mixed ANOVA with repeated measures, two groups and three measurements, α = .05, 1-β = .80, η2 = .06) resulted in a total sample size of 44 participants.^34^ Based on the experience of other studies, a drop-out rate of 15% was assumed.^35^ Thus, 50 participants for the total sample were required. In addition, participation and adherence to physical activity interventions have not been well documented in previous studies,^36^ and a higher drop-out rate in IG has been hypothesized (eg, because of motivational problems); the sample of IG was doubled. A sample size of 75 subjects was included in the study. After inclusion in the study, unfortunately, six legal representatives withdrew their consent and their relatives had to be excluded. Therefore, sixty-nine subjects (58 female/11 male) with dementia participated in the study and were randomized into the IG or CG using block randomization with different group sizes.^33^ Before randomization, subjects were stratified. Stratification was performed using the baseline data of age and cognitive impairment. Subsequently, the subjects were randomized into their respective groups. The exercise instructor and the nursing facility performed stratification. 43 participants were assigned to the IG (86.05 ± 5.98 years) and 26 to the CG (84.03 ± 6.01 years). The maximum group size of the intervention groups was eight subjects, as only two group leaders were on-site for each intervention.^37^ With this number of group leaders, optimal supervision can only be guaranteed with such a group size.^37^

Health status was assessed using a medical questionnaire.

### Music-based exercise program

Since there are no precise guidelines regarding the intervention structure for people with dementia, the WHO and S3 guidelines of the German Society for Psychiatry and Psychotherapy, Psychosomatics and Neurology (DGPPN) were followed for the content, structure and temporal planing of the intervention.^38-40^ Based on these guidelines, a 24-week music-based exercise program was developed, separated into two 12-week interventions with an intermediate test. During the 24 weeks, a multidimensional, music-based exercise program was carried out twice a week for 45 to 60 minutes at the respective cooperation partners with a group size of 6 to 8 people with dementia. For this purpose, the cooperation partners always had a sports or larger room where a minimum of 8 people could train. There were always at least 48 hours between the two exercise sessions. A multidimensional program in this context means that the program includes content on strengthening, coordination and balance, as this can be more effective than a single training program.^16,29^ Overall, the program was conducted at a low to moderate intensity. These intensity forms are also recommended in the guidelines.^38-40^ In addition, the form of intervention is important, especially for people with dementia. The movement program preferably performed with the help of a chair due to the mobility limitations of people with dementia.^41^ Two instructors conducted the exercise sessions with many years of experience in conducting programs with physical activity for people with dementia. In addition to the pure exercises, the study actively used music. The music should be used primarily to increase the mood and motivation of the participants^21^ on the one hand and on the other hand to control movement, eg, to vary the intensity through the tempo of the music. Since music can only have a positive effect if perceived as pleasant,^15^ various music genres and styles were tried out before the study. For this testing, the modified Observed Emotion Rating Scale was used to assess participants’ emotions and reactions to individual pieces of music from the 1940s to the 1980s and classical genres.^42^ In addition to emotions, the music’s tempo was also critical, as the music should be used to control the training interventions. The tempo of a piece of music can dictate the flow of movement and thus positively influence its correct execution.^43^ For this purpose, ranges between 60 and 180 beats per minute (bpm) were tested. The tempo had to be adjusted for faster pieces of music with 160 bpm and more. Based on the results, playlists were created for each intervention session, consisting of music from the 50s to 70s and a tempo between 60 and 160 bpm. Songs were selected mainly from the dementia patients’ youth, as they associated them with positive emotions and remembered them.

During the individual sessions, the respective exercises were attempted to be performed to the rhythm of the music (bpm). The exercise units were executed with balls, gymnastic sticks, rubber rings, scarves and dice, but there were also units without equipment. The structure of each unit was always the same. At the beginning of each session, a 10-minute warm-up was carried out in a sitting and standing position, followed by a 5-minute dance exercise in a sitting position. The choreography of each dance was always kept the same to see if the subjects could remember the flow of the choreography. After the dance, the main part of each session began. This part lasted 30 minutes and was divided into a coordination part (15 minutes) and a strengthening part (15 minutes). In each part, 10 to 15 different exercises were performed. The difficulty level of the coordination exercises was increased in the second intervention phase by introducing additional tasks (eg, handling several balls simultaneously). In the coordination part, it was not always possible to train to the rhythm of the music due to individual exercises. In the coordination part, it was only sometimes possible to train to the rhythm of the music due to individual exercises, as some of the activities were complex and had to be learned first. This also resulted in a lack of attention. Therefore, the rhythm was slightly adjusted. Furthermore, in the coordination and strengthening part no number of repetitions was given, but the exercises was done in rhythm. The instructors controlled the execution of the coordination and strengthening exercises. Participants could take breaks at any time if they needed them. At the end of each session, a 10-minute game was played to encourage interaction and increase fun among the participants. A 5-minute cool-down consisting of stretching and relaxation exercises concluded the training. Since the warm-up, main part and cool-down phases differed in intensity and the tempo of the music was also adjusted in each part. Music in the range of 80-120 bpm was played during the warm-up, 60-100 bpm during the central coordination, 100-160 bpm during the strengthening, and 60-100 bpm during the cool-down phase. In the second intervention phase, a slight adaptation of the exercises occurred according to the experiences from the first phase, for example, too complex exercises were replaced by simpler ones.

The CG did not perform physical exercises and received their usual treatments. They were asked to follow their daily routines and not perform additional exercise activities.

## Instruments and Procedure

In order to investigate the study’s objectives, test procedures were used, which, as has been shown in various studies, can also be performed in dementia patients and provide the corresponding information. A variety of test procedures were used since it was shown that, especially in dementia, motor and cognitive functions are affected very individually or deteriorate to different degrees.^44^ For example, it was shown that cognitive function includes multiple subdomains, and it is recommended to examine the individual subdomains.^45^ Therefore, different motor function and cognition subareas have to be examined in such an intervention.^44^ To prevent overwhelming dementia patients with the number of test procedures used, the patients performed the motor tests on one day and the cognitive tests on another. In addition, the Dementia Mood-Picture Test^46^ was used to assess mood and acceptance of the intervention among the subjects. The Dementia Mood-Picture Test consists of six faces showing moods ranging from happy to bad. Subjects were asked to indicate their mood using the pictures.^46^ This test was performed before and after each training session.

### Motor Tests and Outcome Parameters

All used motor test procedures were also recommended for people with dementia in the work of Trautmann et al^47^ Data collection of motor performance always began with the hand dynamometer test to determine grip strength.^48^ The hand dynamometer test was performed with each subject three times with both hands. The best value of each hand was included in the evaluation. The hand dynamometer test provides information about overall strength ability.^49^ Afterward the drop-bar test followed to determine motor reaction time.^50^ It was performed with the previously determined dominant hand three times on each subject. Again, the best value was included in the evaluation. The modified chair-rising test followed to assess lower extremity strength ability.^51^ This test requires subjects to stand up and sit down five times as quickly as possible while using arms is allowed. The required time is measured. It provides information about the subjects’ leg strength and risk of falling.^51^ The FICSIT-4 was then performed to assess balance.^52^ This test consists of four positions (bilateral, semi-tandem, tandem, and unilateral), performed with open and closed eyes.^52^ Time is measured and converted to a point scale (maximum 28 points). Finally, the timed-up-and-go-test was conducted to assess mobility.^53^ This test required subjects to stand up from a chair, walk 3 meters, make a 180-degree turn, walk back, and sit down again.^53^ The time it took subjects to complete the task was measured. For the modified chair-rising, drop-bar, and timed-up-and-go-test, lower scores correspond to better performance, while higher scores indicate better performance for the hand dynamometer and FICSIT-4 test. A detailed description of the test procedures and the quality criteria are attached to Supplement 1.

### Cognitive Tests and Outcome Parameters

To examine cognitive abilities, the Cerad-NP-Plus (Consortium to Establish a Registry for Alzheimer’s` Disease) was conducted.^54^ This questionnaire was developed specifically for dementia patients. It consists of ten subtests that examine different cognitive domains, which can be found in Table 1. Word List Saving comprises the subtests Word List and Word List delayed recall. The same applies to constructive practice saving, consisting of constructive practice copy and constructive practice recall. Discriminability is calculated from the scores achieved in the Recognition recall subtest. A higher score corresponds to better performance for every subtest except the Trail-Making Test-A and B. In addition to the raw scores of each test procedure, the z-scores of the CERAD-NP-Plus were calculated. The calculated z-values are based on a complex normalization formula, which ensures that the obtained z-values are extremely precise and have good mathematical properties.^55^ A z-value of zero would thus correspond precisely to the average of the norm population. A positive value means the patient’s performance is above the average, whereas a negative value corresponds to a performance below the average.^55^ A detailed description of the test procedures and the quality criteria are attached to Supplement 1.

**Table 1.** Cognitive test procedures.

CERAD-NP-Plus (Morris et al 1989)		
Subtest	Cognitive area	Description
Verbal fluency (animals)	Executive functions	-name as many animals as possible in one minute
Boston-naming-test	Word finding and naming, visual perception	-The short form contains 15 images that would need to be named
Mini-Mental- state-Examination	General cognitive function level (screening)	-Answering questions to determine the cognitive abilities of older people
Word list	Verbal memory	-Ten words are read out one after the other and then recalled from memory -is performed three times, are the same ten terms only in different orders
Constructional practice copy	Visuoconstructive skills	-4 pictures have to be painted (circle, parallelogram, 2-square, cube)
Word list delayed recall	Verbal memory (delayed verbal memory)	-Free reproduction of the ten terms from the word list memory test
Word list Recognition recall	Verbal memory (delayed verbal memory, recognition)	-The ten already mentioned terms and ten new terms are presented to the respondent -The respondent has to declare the ten already mentioned terms as “already known” or “not known.”
Constructional practice recall	Nonverbal memory (delayed figural memory)	-Drawing the figures from the constructive part of memory
Trail-making Test-A	Psychomotor speed, executive functions	-Number linking as fast as possible (from 1-25)
Trail-making Test-B	Executive functions	-Combine numbers and letters alternately, keeping the numerical and alphabetical sequence, respectively (1-13; A-L)

### Quality of Life

Quality of life (QoL) status was assessed using the Qualidem.^56^ The QoL instrument was developed specifically for dementia patients and consists of nine dimensions: Care relationship, positive affect, negative affect, agitated, tense behavior, positive self-image, social relationships, social isolation, feeling at home, and having something to do. Higher scores indicate higher quality of life for the person with dementia. The responsible nursing staff assessed the QoL. A detailed description of the test procedures and the quality criteria are attached to Supplement 1.

### Statistical data Analysis

Data from the Dementia-Mood-Picture Test were analyzed qualitatively. Statistical data analysis was performed using SPSS, version 28 (IBM). All quantitative variables were indicated as mean ± standard deviation. Group differences at baseline (age, height, weight, body mass index (BMI)) were compared with a t-test or chi-square-test. A mixed ANOVA with repeated measures was conducted with the between-subjects factor group and the within-subjects factor time. The group effects (group) were irrelevant for analyzing the questions but can be found in Supplement 2.

When the ANOVA revealed a significant effect, post-hoc analyses were performed with a Dunn-Bonferroni correction. Mixed ANOVA was also used in case of violation of normal distribution and emerged outliers since previous studies have already shown its robustness.^57^ The significance level was set to α = .05. Cohen’s classification^58^ was used to interpret the effect sizes (η_p_^2^ = .01/*f =* .1, small; η_p_^2^ = .06/*f =* .25, moderate; η_p_^2^ = .14/*f =* .4, large).

## Results

### Sample

**Figure 1.**
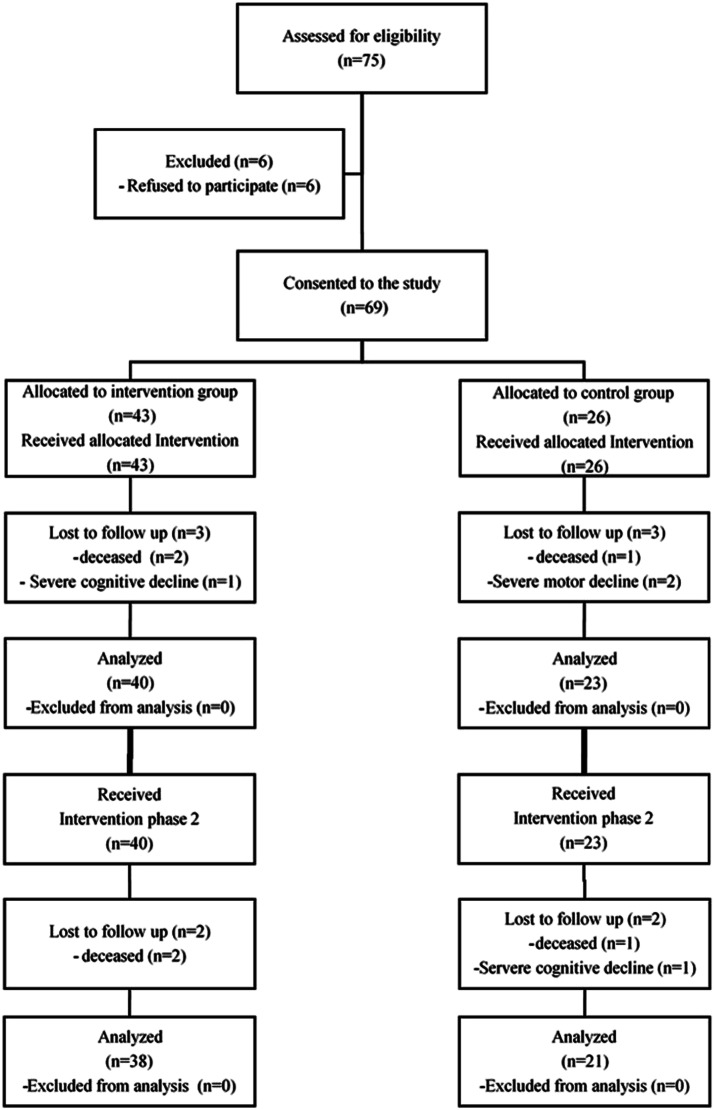
Trial-flow Chart.

Six subjects dropped out during the first (IG = 3 and CG = 3), and four during the second intervention phase (IG = 2 and CG = 2) (Figure 1). This corresponds to a drop-out rate of 11% in the IG and 19% in the CG. The reasons were cognitive and motor decline (becoming bedridden or dependent on a wheelchair) or passed away. In the case of predominant bedriddenness or permanent wheelchair use, the intervention has not been possible, and thus they have been excluded. At the end of the study, a total of fifty-nine subjects, 38 from the IG (age: 84.05 ± 5.73 years) and 21 from the CG (age: 83.71 ± 6.34 years), were included in the analysis (see Figure 1).

### Baseline Characteristics

Table 1 shows the baseline data of the subjects from each group. More women participated in both the IG and the CG (IG = 84.8%, CG = 87.5%) and participants of the IG had an overall attendance of 93% in all exercise sessions. The degree of dementia shows that two subjects had no dementia, and four had severe dementia. These data were collected by the screening method Mini-Mentel-State-Examination. Especially in the case of moderate and severe dementia, the MMSE is sometimes inaccurate.^59^ May be classified with severe dementia according to the MMSE, although they could still carry out the exercise program without problems. All baseline data were statistically examined using the t-test or the chi-square test. For the degree of dementia and the type of dementia, the percentage values were used for statistical analysis. No statistically significant differences (*P* > .05) were found between groups at baseline (see Table 2).

**Table 2.** Baseline characteristics.

Characteristics	Intervention group (n = 38)	Control group (n = 21)	*p-Value*
	M ± SD	M ± SD	
Age (years)	84.05 ± 5.76	83.71 ± 6.34	.789^t^
BMI	27.23 ± 5.09	27.35 ± 4.82	.433^t^
Sex	m: 15.2% f: 84.8%	m: 12.5% f: 87.5%	.804
MMSE score	18.61 ± 5.8	18.10 ± 7.04	.380^t^
Education years	11.05 ± 3.23	9.86 ± 2,67	.075^t^
Attendance at practice sessions	93%	/	
Degree of dementia			
No dementia	1 (2.6%)	1 (4.8%)	1.0
Mild dementia	17 (44.7%)	10 (47.6%)	.397
Moderate dementia	19 (50%)	7 (33.3%)	.400
Severe dementia	1 (2.6%)	3 (14.3%)	.261
Forms of dementia			
Alzheimer’s dementia	22 (57.9%)	10 (47.6%)	.408
Vascular dementia	3 (7.9%)	2 (9.5%)	.287
Other types of dementia	13 (34.2%)	9 (42.9%)	.382

*Notes.* **M** Mean values. **SD** standard deviations. **p** significance <.05. **m** male. **f** female. **^t^** t-test.

### Outcome Parameters

The Trail-Making-Test-B could only be performed by a few subjects due to its complexity. It was unsuitable for our dementia and therefore removed from the statistical analysis. The Dementia Mood Picture Test showed that subjects were more likely to be in a good mood after the interventions. However, 15% of the subjects were in a bad mood or felt anxious before the interventions. After the intervention, this number decreased to 5%.

The means and standard deviations of the primary and secondary outcomes are shown in Table 3. At baseline (T0), no statistically significant differences were found between the IG and CGs in motor parameters (handgrip strength right/left, drop-bar-test, modified chair-rising-test, FICSIT-4, timed-up-and-go), cognitive parameters and quality of life (*P* > .05 for all).

**Table 3.** Results from baseline (T0), intermediate (T1) and post-test (T2) for the intervention group and control group (mean (M) ± standard deviation (SD)).

Test procedure	Intervention group n = 38			Control group n = 21		
	T0 M ± SD (Cl_95_)	T1 M ± SD (Cl_95_)	T2 M ± SD (Cl_95_)	T0 M ± SD (Cl_95_)	T1 M ± SD (Cl_95_)	T2 M ± SD (Cl_95_)
Motor performance						
Hand grip strength right (N)	154.9 ± 76.2 (129.8-179.9)	167.6 ± 77.4 (142.2-179.9)	172.4 ± 77.9 (146.8-198.0)	144.1 ± 61.9 (115.9-172.2)	133.2 ± 58.9 (106.4-159.9)	110.9 ± 55.8 (85.6-136.4)
Hand grip strength left (N)	134.3 ± 61.2 (114.2-154.4)	155.2 ± 70.3 (132.1-178.3)	159.9 ± 70.8 (136.7-183.2)	133.7 ± 56.4 (108.0-159.3)	132.5 ± 53.9 (107.9-157.1)	112.4 ± 58.4 (85.8-138.9)
Drop-bar-test (cm)	25.9 ± 11.4 (22.1-29.7)	20.4 ± 10.1 (17.0-23.8)	19.1 ± 6.9 (16.8-21.4)	26.1 ± 11.3 (20.9-31.3)	29.1 ± 13.2 (23.2-35.1)	27.8 ± 15.1 (21.4-34.2)
Modified Chair-rising-test (s)	17.6 ± 9.7 (14.4-20.8)	15.5 ± 8.0 (12.8-18.2)	15.4 ± 9.5 (12.2-18.6)	20.4 ± 6.9 (17.1-23.6)	19.5 ± 6.6 (16.4-22.6)	24.5 ± 9.9 (19.8-29.1)
FICSIT-4 (s)	16.6 ± 8.2 (13.9-19.2)	16.9 ± 7.9 (14.4-19.5)	17.9 ± 6.8 (15.7-20.2)	13.4 ± 6.1 (10.6-16.2)	11.7 ± 6.6 (8.7-14.7)	9.9 ± 5.8 (7.3-12.6)
Timed-up-and-go-test (s)	20.1 ± 12.1 (16.1-24.2)	16.0 ± 10.3 (12.6-19.5)	15.5 ± 9.5 (12.4-18.7)	24.4 ± 6.7 (21.3-27.5)	23.5 ± 7.8 (19.8-27.2)	28.3 ± 10.9 (23.1-33.7)
Cognition performance						
Verbal fluency (max. number)	11.5 ± 6.2 (9.4-13.5)	12.8 ± 5.9 (10.9-14.7)	13.7 ± 6.4 (11.6-15.8)	10.9 ± 6.7 (7.9-14.0)	10.1 ± 5.8 (7.5-12.8)	8.2 ± 5.5 (5.8-10.7)
Bosten-naming-test (max. 15 points)	9.6 ± 3.2 (8.5-10.7)	10.2 ± 3.0 (9.2-11.2)	10.1 ± 3.1 (9.1-11.2)	9.4 ± 4.3 (7.4-11.3)	9.1 ± 3.6 (7.5-10.8)	7.9 ± 4.5 (5.8-9.9)
MMSE (total points)	18.6 ± 5.8 (16.7-20.5)	18.6 ± 5.7 (16.8-20.5)	18.6 ± 5.4 (16.8-20.4)	18.6 ± 5.4 (16.8-20.4)	16.9 ± 7.0 (13.7-20.1	15.1 ± 7.3 (11.8-18.4)
Wordlist Saving (%)	27.7 ± 37.6 (15.4-40.1)	30.8 ± 31.1 (20.6-41.0)	31.9 ± 30.4 (22.0-41.9)	31.9 ± 30.4 (22.0-41.9)	29.7 ± 35.7 (13.4-45.9)	29.6 ± 45.0 (9.1-50.1)
Discrimina-teability (%)	71.9 ± 20.4 (65.3-78.7)	73.2 ± 15.9 (67.9-78.4)	72.1 ± 18.0 (66.2-78.0)	72.1 ± 18.0 (66.2-78.0)	69.1 ± 22.2 (58.9-79.2)	61.4 ± 23.2 (50.9-72.0)
constructive Practice saving (%)	14.0 ± 25.6 (5.6-22.4)	11.8 ± 22.9 (4.2-19.3)	6.8 ± 14.1 (2.2-11.5)	6.8 ± 14.1 (2.2-11.5)	19.6 ± 28.9 (6.4-32.8)	16.1 ± 28.9 (3.0-29.3)
Trail-making-test-A (s)	143.3 ± 80.1 (114.5-172.2)	133.4 ± 70.6 (108.0-158.9)	132.3 ± 69.9 (107.1-157.4)	132.3 ± 69.9 (107.1-157.4)	170.0 ± 81.9 (127.9-212.1)	189.4 ± 81.0 (147.8-231.1)
Measurement of quality of life						
Qualidem (total points max.240)	183.5 ± 25.1 (175.3-191.8)	184.2 ± 24.6 (176.1-192.3)	191.4 ± 21.5 (184.4-198.5)	181.0 ± 32.5 (166.2-195.8)	181.1 ± 30.0 (167.4-194.8)	171.7 ± 36.0 (155.4-188.1)

*Note***. FICSIT-4** Frailty and Injuries: Cooperative Studies of Intervention Techniques. **MMSE** Mini-Mental-State Examination. **CL**_**95**_ 95% confidence interval.

Subsequently, the results were analyzed using a mixed ANOVA with repeated measures, regarding the interaction effect between the groups (time*group).For a better interpretation of the interaction effect, the within-group effects were additionally analyzed. The between-subject effects (group) were irrelevant for analyzing the questions but can be found in Supplement 2.

When analyzing the interaction effects between the intervention and control groups, significant results were found in the motor and cognitive test procedures and the quality of life. There was a significant interaction effect for all selected motor skills at time T2, after 24 weeks (see Table 4). A significant interaction effect was demonstrated for the cognitive abilities, particularly in verbal fluency, the Bosten naming test, the MMSE, and the Trail-Making Test A (see Table 4). These also occurred primarily after 24 weeks (T2). No significant difference was found in the other cognitive tests. In quality of life, a significant interaction effect could also be determined at T2 (see Table 4).

**Table 4.** Results of the comparison of the interaction and within of the intervention (IG) and control (CG) group.

Test procedure	Time*group effects				Within-group effects		
	F (df)	*p* (T0,T1,T2)	Effect size η_p_^2^		F (df)	*p*	Effect size *f*
Modified chair-rising-test	F(2,114) = 13.035	<.001 (T2:.001)	.194	IG	F(2,74) = 10 873	<.001^a,b^	.56
				CG	F(2,40) = 6357	.004^b^	.58
Drop-bar-test	F(2,114) = 5.350	.006 (T1:.006) (T2:.003)	.090	IG	F(2,74) = 12 952	<.001^a,b^	.61
				CG	F(2,40) = 0.500	.646	.16
Hand grip strength right	F(2,114) = 15.706	<.001 (T2:.002)	.225	IG	F(2,74) = 7166	<.001^b^	.44
				CG	F(2,40) = 7638	.002^b^	.62
Hand grip strength left	F(2,114) = 24.207	<.001 (T2:.011)	.310	IG	F(2,74) = 30 230	<.001^a,b^	.90
				CG	F(2,40) = 6036	.005^b^	.55
FICSIT-4	F(2,114) = 10.728	<.001 (T1:.012) (T2:.001)	.166	IG	F(2,74) = 3216	.05	0.3
				CG	F(2,40) = 6307	.004^b^	.58
Timed-up-and-go- test	F(2,114) = 14.430	<.001 (T1:.007) (T2:.001)	.211	IG	F(2,74) = 20 787	<.001^a,b^	.77
				CG	F(2,40) = 4797	.014^c^	0.5
Verbal fluency	F(2,114) = 22.626	<.001 (T2:.002)	.284	IG	F(2,74) = 14 913	<.001^a,b^	.63
				CG	F(2,40) = 9039	<.001^b,c^	.67
Bosten-naming-Test	F(2,114) = 10.396	<.001 (T2:.026)	.154	IG	F(2,74) = 2466	.092	.26
				CG	F(2,40) = 13 730	<.001^b,c^	.83
MMSE	F(2,114) = 12 643	<.001 (T2:.039)	.182	IG	F(2,74) = 0.003	.997	0
				CG	F(2,40) = 19 934	<.001^b,c^	.99
Wordlist saving	F(1,57) = .003	.956	.00	IG	F(2,74) = 0.517	.593	.12
				CG	F(2,40) = 0.108	.893	.07
Discriminate -ability	F(1,57) = 3.194	.051	.053	IG	F(2,74) = 0.125	.883	.05
				CG	F(2,40) = 5640	.007^b^	.53
Constructive practice saving	F(1,57) = 3.332	.073	.055	IG	F(2,74) = 3813	.069	.32
				CG	F(2,40) = 5439	.008^a,b^	.52
TMT-A	F(2,114) = 18.043	<.001 (T2:.013)	.277	IG	F(2,74) = 5122	.009^a,b^	.41
				CG	F(2,40) = 13 541	<.001^b,c^	.92
Qualidem	F(2,114) = 12.382	<.001 (T2:.011)	.178	IG	F(2,74) = 7224	.001^b,c^	.44
				CG	F(2,40) = 5544	.007^c^	.53

*Note.* **FICSIT-4** Frailty and Injuries: Cooperative Studies of Intervention Techniques. **MMSE** Mini-Mental-State Examination. **TMT-A** Trail-Making-test-A. **df** degrees of freedom.

^a^Within-group effects: significantly different between T0-T1 (statistically significant: *P* ≤ .05).

^b^Within-group effects: significantly different between T0-T2 (statistically significant: *P* ≤ .05).

^c^Within-group effects: significantly different between T1-T2 (statistically significant: *P* ≤ .05).

**Figure 2.**
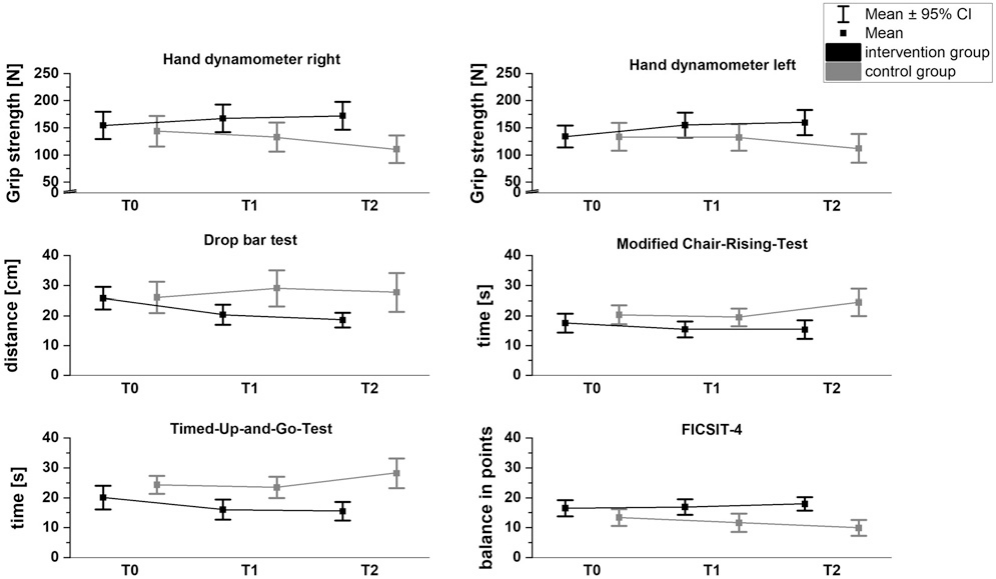
Time*group effects for the motor parameters. Note*. Higher values in the hand dynamometer and FICSIT-4 signal better performance, whereas, in the Drop bar test, Timed-Up-and-Go-test and Modified Chair-Rising-test, lower values are better.*

**Figure 3.**
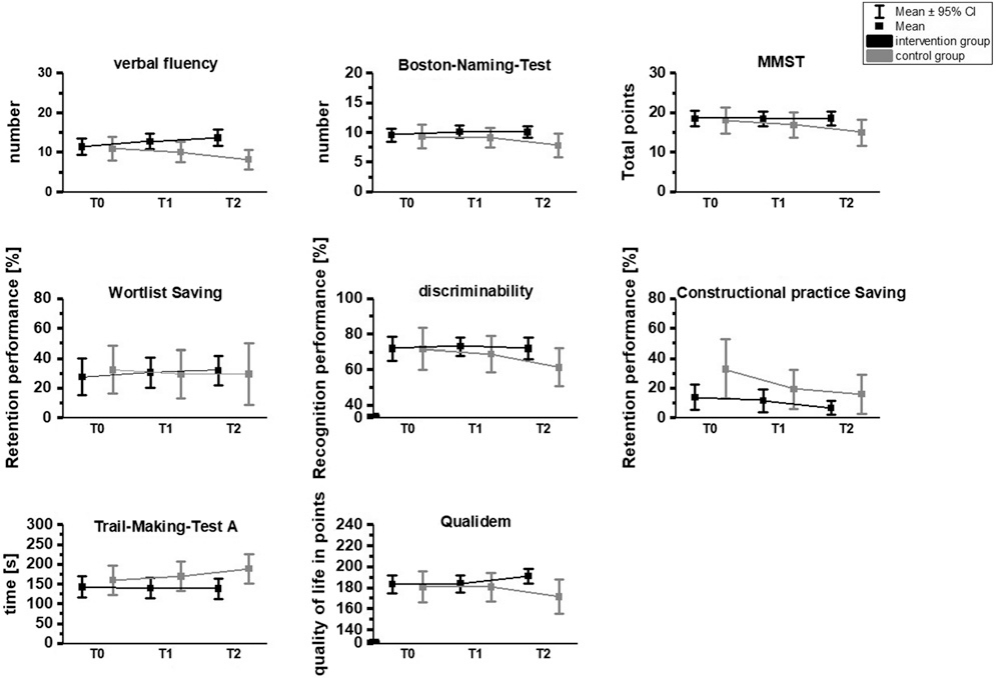
Time*group effects for cognitive parameters and quality of life. Note. *Higher scores signal better performance for all cognitive tests and the quality of life, except for the Trail-Making-Test A, where a lower score indicates a better result.*

The significant interaction effects occurred with a large effect.^58^ For clarity, the interactions between the groups were visualized in Figures 2 and 3.

Based on the descriptive values from Tables 3 and it can be seen that the IG improved over time compared to the control group. However, to prove this statistically and better interpret the interaction effects, the within-group effects were also analyzed.

In the modified chair-rising-test (T0-T1, *P* = .003; T0-T3, *P* = .002), drop-bar-test (T0-T1, *P* < .001; T1-T2, *P* < .001), handgrip strength left (T0-T1, *P* = .001; T1-T2, *P* < .001) and timed-up-go test (T0-T1, p<. 001; T0-T2, *P* < .001), the IG improved significantly after three and six months of intervention compared to the baseline test. In the handgrip strength right (T0-T2, *P* = .008), a significant improvement was only observed after the 6-month intervention. In the FICIST-4, the IG showed no significant changes. In contrast, the CG showed significant deterioration in motor skills throughout the study. In the modified Chair-Rising-Test (T0-T2, *P* = .045), Handgrip strength right (T0-T2, *P* = .017) and left (T0-T2, *P* = .04) and FICSIT-4 (T0-T2, *P* = .015), the CG deteriorated significantly after six months. In the timed-up-and-go test, they worsened significantly from T1 to T2 (*P* = .038) (see Table 4). No significant change was found in the drop-bar test. However, all significant differences occurred with a large effect.^58^

Similar results were found for cognitive skills (see Table 4). The IG significantly improved in verbal fluency (T0-T1, *P* = .005; T0-T2, *P* < .001) and Trail-Making-Test-A (T0-T1, *P* = .047; T0-T2, *P* = .031) after both 3- and 6-month. No significant change over time could be found in the IG in the other cognitive parameters. In the CG, significant deteriorations were found in verbal fluency (T0-T2, *P* = .009; T1-T2, *P* = .003), Bosten naming test (T0-T2, *P* < .001; T1-T2, *P* = .003), Mini-Mental State Examination (T0-T2, *P* < .001; T1-T2, p = . 001), discriminability (T0-T2, *P* = .026), constructive practice saving (T0-T1, *P* = .027; T0-T2, *P* = .047) and Trail-Making-Test-A (T0-T2, *P* < .001; T1-T2, *P* = .002) over 3- and 6- months. No significant change was detected in the CG for the word list saving. All significant differences occurred with a large effect.^58^ In terms of quality of life, the IG improved from T0 to T2 (*P* = .014) and T1-T2 (*P* = .004), whereas the CG significantly worsened from T1-T2 (*P* = .009). The significant changes occurred with large effects.^58^

Similar interactions can also be seen in the normalized results of the CERAD-NP-Plus (z-scores) (see Supplement 3). Here, a deterioration of the CG and an improvement of the IG after 6 months can be observed (see Supplement 3).

## Discussion

Every person wants to experience a pleasant, dignified old age For people with dementia, however, this is not always possible due to the disease; for example, the risk of falling is increased, and thus independence is limited. There is evidence that combining music and physical activity can stabilize the cognitive and motor performance of people with dementia, as these abilities are receptive through music or physical activity until the late phase.^25^

Especially for maintaining independence, the preservation of motor performance is necessary to a certain degree, but this is also influenced by cognitive performance. For that reason, it is essential to train these two performance factors together and stabilize both. Therefore, the present study aimed to investigate the influence of an 24-week multidimensional music-based exercise program on selected motor and cognitive performance and quality of life of dementia patients. For this purpose, an intervention group that performed the described exercise program was compared with a passive control group that did not complete any additional intervention at three measurement time points (T0, T1 (after 12 weeks), and T2 (after 24 weeks). First, it was found that this form of intervention could be implemented with dementia patients and was positively received.

Furthermore, this study shows that a multimodal approach consisting of training several skills and abilities can improve selected motor and cognitive performance and quality of life compared to no intervention. These results confirm the statements from comparable studies. For example, the results of Borges-Machado et al^29^ show that a multidimensional training program can be implemented in dementia patients and has positive effects. This study also demonstrated that music could be integrated and increases motivation and training intensity.

In our study, it was shown that a chair-based intervention is particularly suitable for dementia patients with limited mobility and produces positive effects. This finding is supported by Cordes et al^60^ and Cordes et al,^41^ who studied this form of intervention with a primary focus on its usage in a long-term care setting. For example, in an elastic band training, Chen et al^61^ examined handgrip strength in older adults with cognitive impairment and found a significant improvement. Hand strength is an indicator of well-being and overall strength^49^ and also increased after our music-based training.

Blankevoort et al^62^ demonstrated that lower limb strength improved equally through multicomponent interventions and progressive resistance training. In our study, lower limb strength also improved (modified chair-rising test) Blankevoort et al^62^ also found a similar effect on mobility (timed-up-and-go test), which also improved in our study after the 24-week intervention. In addition, we observed interaction effects in all motor domains, indicating that the IG improved significantly compared to the CG at the end of the intervention. Mobility (modified chair-rising test, timed-up-and-go test) and balance (FICSIT-4) are particularly noteworthy, as these are critical factors in maintaining independence.^63^ In the cognitive domain, significant improvements were found in verbal fluency and executive functions. Brancatisano et al^7^ and van de Winckel et al^64^ found similar effects for their music-based exercise programs. The improvement in executive functions may occur since an active musical intervention, which includes the music-based movement program, is more likely to promote socialization, engagement, verbal processing, or motor planning than a receptive musical intervention.^45^ They also observed significant improvements in MMSE scores.^7,64^ However, the results of the present study only descriptively show a slight improvement in the MMSE score after the intervention. Still, the results of the MMSE should always be viewed with caution, as several studies have shown that the MMSE score improves with a music-based intervention but, for example, the Montreal Cognitive Assessment Score (MOCA) does not improve, even though they both have global cognition tests.^45^ Cheung et al^30^ were also able to observe an improvement or influence of cognitive abilities after music-with-movement intervention over time. Nevertheless, at the same time, they showed that there was no difference between different active groups. Therefore, it is also important to note that the comparison group is either another active intervention group or an inactive control group, especially in terms of cognitive skills. The improvement in cognitive performance may also be because the program included motor and cognitive tasks, which may positively affect cognitive performance.^65^

However, our results show interaction effects in the cognitive domain because the CG significantly deteriorates in almost all cognitive parameters over the study, whereas the IG improves or stabilizes in many. In particular, the stabilization of cognitive abilities over six months can be considered equally positive, as usually, a continuous decline in cognitive abilities over such a period is expected in dementia.^66,67^ Toulotte et al^66^ demonstrated that people with dementia experience a loss of function within three months without intervention. However, a passive control group could also lead to problems because, on the one hand, a placebo effect could arise.^45^ On the other hand, one cannot judge whether the positive effects are due to the music-based movement intervention or the pure music or the pure movement. Participants in such an intervention group might think they receive a positive intervention/treatment.^45^ Therefore, a placebo effect might occur because they expect their cognitive and motor functions to improve. Therefore, future studies should use an active control group to reduce the placebo effect.^45^ These active control groups could then receive a pure music and movement intervention, allowing more accurate conclusions about the effect.

As already described, a change in cognitive abilities also affects motor function as cognitive ability decisively influences motor performance.^63,68^ Similar tendencies of the positive effect of the intervention could be shown within the quality of life. The quality of life improved in the IG, whereas it worsened in CG. Similar results can be found in van Steen et al^69^ and Henskens et al^70^ In contrast, Ojagbemi and Akin-Ojagbemi^71^ could not notice any effects on quality of life.^71^ These conflicting statements could arise because assessing the quality of life of people with dementia is complex. Furthermore, it is known that proxy ratings of quality of life are influenced by the level of distress or state of the emotional well-being of the rater.^72^

Overall, the significantly interaction effects, especially after six months. After this period, the CG deteriorates, whereas the IG improves, showing that the length of the intervention has a decisive influence on the effects on motor and cognitive performance. However, an important consideration is whether the intervention's duration might compromise the music-based intervention's beneficial effect, particularly on cognitive functions.^45^ Some studies have shown that short-term interventions also positively affect cognition.^45^ Therefore, further studies should examine the effects of a shorter and longer-term music-based intervention and, in particular, the focus on a follow-up test.

In addition to physical activity, music may have influenced the results. For example, people with dementia are receptive to music, which positively affects their mood and behavior.^7,14^ However, it should be noted that music can only have a positive effect if it is familiar and perceived as pleasant.^15,73^ Therefore, each subject should have the opportunity to listen to their preferred music. Different music for each subject should also be possible in group training but is difficult to manage and currently not feasible. However, the exercise program should always be offered as group training to promote interaction and social components. Another positive effect is that dementia patients are not isolated.^6^

Another factor that may have led to the positive effects is the communication used during the intervention. For dementia patients, communication is of great importance and can create a feel-good atmosphere in which they feel more comfortable and perform the exercises better.^74^ Communication was always structured and straightforward and should always support and motivate the subject during the training.^74^ This should also be considered in subsequent studies.

In summary, a music-based multidimensional exercise program was developed, which on the one hand, was feasible in people with dementia and, on the other hand, improved or stabilized both motor and cognitive skills as well as the quality of life. Additionally, compared to a passive CG, it could be shown that the music-based multidimensional exercise program as a form of non-pharmacological therapy could counteract the decline of motor and cognitive abilities.

However, the facilities must also implement or use these potentially effective programs. It has already been shown that it takes an average of 17 years for evidence-based practices to be incorporated into routine general health care practice.^75^ To shorten this, the key to success lies in working closely with external stakeholders with good communication skills and understanding cultural perspectives and human factors.^75^ Likewise, further studies should also focus on implementing science to accelerate the integration of research innovations into practice.^75^ Particularly against the Corona pandemic backdrop, this again takes on a high significance. However, due to an even lower activity level and isolation during the Corona pandemic, the physical performance of people with dementia has deteriorated again. It shows that, especially in this target group, more needs to be done or studied.^76^

Based on these results, our first hypothesis can be accepted that a music-based multidimensional movement program can improve and maintain motor and cognitive functions. The second hypothesis that the intervention can improve or maintain quality of life can also be accepted because improvements in Qol instruments were detected after the intervention. However, this needs to be further investigated in upcoming studies, as this study also had some limitations.

### Limitations

There are some limitations of this study that should be considered in future work. The multimodal approach should be applied to larger groups to confirm the results. Block randomization with equal-sized groups would be preferable in future studies, as better statistical power would allow more accurate conclusions in this case. Also, to minimize potential bias, in addition to increasing statistical power, an intention-to-treat analysis should be performed for subsequent RCTs. This was not investigated in more detail in this pilot study. In addition, further studies should reconsider the selected testing procedures. A framework by Cheung et al^77^ (2011) shows that interventions that can reduce stress can lower anxiety and depression levels, subsequently reduce agitation, and improve cognitive function in people with dementia. Therefore, additional tests that measure stress, anxiety, and depression should be added to improve the interpretation and validity of cognitive function. Special consideration should also be given to the preferences of the person with dementia regarding the intervention’s content or format, such as the type of music and cultural preferences.Otherwise, stress may be induced. The Dementia-Mood-Picture-Test is unsuitable for patients with moderate to severe dementia, as its validity must be doubted in people with dementia with increasing cognitive impairment.Thus, other test procedures should be used to assess the mood,^78^ such as the Observation Emotion Rating Scale.^42^

Furthermore, the calculation of a sum score across all subscales in the Qualidem must be viewed critically. However, this was done for methodological and statistical reasons.^79^ In subsequent studies, the nursing staff should also be blinded to the quality of life survey. Quality of life is assessed with questionnaires, which the nursing staff subjectively rates. In this respect, the data could be biased if it is known who belongs to which group. Likewise, the test environment must be considered. The tests were carried out on the facilities of the cooperation partners, and thus identical test environments were not always given. However, cognitive performance can mainly be influenced by different factors like the test environment (room, temperature, etc.), motivation, or mood.^55^ Therefore, in subsequent studies, test environments should be chosen that contain few variable stimuli.^55^

In the future, the duration and intensity of the training programs should be differentiated according to dementia levels or severity.^80,81^ On the one hand, this must be done because of a better grouping and the associated equal starting level of the test subjects within the groups and, on the other hand, to avoid overload or underload.^80,81^ To better address this problem, it is important to draft initial guidelines for training dementia patients that provide information about the intensity, duration and form of intervention. In addition, in following studies dementia severity should not be assessed using the MMSE, as it is a screening test for assessing the degree of dementia. Furthermore, it should be viewed critically, as it sometimes provide unusable values for moderate and severe dementia.^59^ In order to obtain more precise information about the degree of dementia, neurologists would have to be consulted, or the Global Deterioration Scale could be used.^30^

In addition to dividing the groups according to the degree of dementia, the group size also plays a crucial role. As mentioned earlier, the group size is an essential factor for interaction and dynamics. Therefore, the exercise program should be tested in follow-up studies with larger groups (n < 15). However, more support staff is needed to implement the program. In addition to the degree of dementia and group size, follow-up studies must consider group composition and homogeneity. For example, in this study, over 80% of the participants were female. Therefore, more male dementia patients should be included to make generally valid statements. Furthermore, future studies should examine the clinical relevance, besides statistical significance.^82^ Then, conclusions could be drawn about the effectiveness of the exercise program, especially for everyday life. Furthermore, dual-task exercises should be increasingly integrated into such aprogram. These tasks are particularly useful for fall prevention, as impaired cognitive abilities lead to insufficient compensation of sensory dysfunctions and thus increase the risk of falls.^83^ In addition, inclusion and exclusion criteria should be refined. The hearing ability needs to be considered, it is crucial in music-based programs. However, especially in this age structure and dementia patients hearing is limited, which is why alternatives must be found so everyone can hear and feel the music.^84^ The duration of the intervention and the number of training sessions should also be reconsidered. For organizational reasons, the intervention in this study was conducted twice a week for 60 minutes over 12 weeks. Guidelines recommend longer intervention duration and more frequent training sessions for sustained effects.^38^ The small Corona pandemic-related interruption of 2 to 3 weeks may have influenced the results as well. Mainly due to the pandemic and the resulting isolation of the residents, a rapid reduction in performance may have taken place.^85^

## Conclusion

A multidimensional music-based exercise program for dementia patients was developed and tested, targeting strength, endurance, coordination, and balance. The exercise program was well received by people with dementia and showed positive results. In addition to the positive results, no significant deterioration was observed in any of the test procedures, and thus stabilization or resource preservation was achieved. This can be considered positive in the case of dementia. Furthermore, the results indicate that the applied intervention can improve the parameters relevant to maintaining independence more sustainable than usual care. However, this should be further investigated in subsequent randomized controlled trial studies, considering and compensating for the limitations identified in this study. Finally, after a further investigation based on this study, it could be concluded that a multidimensional music-based exercise program can serve as a non-pharmacological alternative or adjunct to drug therapies.

## Supplemental Material

Supplemental Material - Changes in Selected Cognitive and Motor Skills as Well as the Quality of Life After a 24-Week Multidimensional Music-Based Exercise Program in People With Dementia Click here for additional data file. Supplemental Material for Changes in Selected Cognitive and Motor Skills as Well as the Quality of Life After a 24-Week Multidimensional Music-Based Exercise Program in People With Dementia by Alexander Prinz, Anneke Schumacher and Kerstin Witte in American Journal of Alzheimer's Disease & Other Dementias®

## Data Availability

The data given this article are the data sets and protocols generated and analyzed in the current study are available from the corresponding authors upon request.
