# Supplementary material for: Changes in Selected Cognitive and Motor Skills as Well as the Quality of Life After a 24-Week Multidimensional Music-Based Exercise Program in People With Dementia
Source: Am J Alzheimers Dis Other Demen. 2023 Aug 23;38:15333175231191022. doi: 10.1177/15333175231191022 (PMC10655793; doi:10.1177/15333175231191022)
Supplement: Supplemental Material - Changes in Selected Cognitive and Motor Skills as Well as the Quality of Life After a 24-Week Multidimensional Music-Based Exercise Program in People With Dementia [file sj-pdf-1-aja-10.1177_15333175231191022.pdf]

Table 1

cognitive test procedures

**cognition**		
**CERAD-NP-Plus** (Morris et al., 1989)		
**Test procedure (subtest)**	**Cognitive area**	**Declaration**
verbal fluency (animals)	Executive functions	- - name as many animals as possible in one minute<br>- - Score is unlimited
Boston-Naming-Test	Word finding and naming, visual perception	- the short form contains 15 images that would need to be named<br>- 3 groups of items:<br>o first group contains 5 frequent items<br>o the second group contains 5 medium frequent items,<br>o the third group contains 5 rare items
Mini-Mental State Examination	General cognitive function level (Screening)	- Answering questions to determine the cognitive abilities of older people<br>- - maximum 30 points
Memory list sum of immediate recall	Verbal memory	- Ten words are read out one after the other and then recalled from memory.<br>- - is performed three times, are the same ten terms only in different orders<br>- - Number of remembered terms in the three rounds á ten terms (max. 30 points possible)
Constructional praxis copy	Visuoconstructive skills	- 4 pictures have to be painted (circle, parallelogram, 2-square, cube)<br>- - maximum 11 points
Word list delayed recall	Verbal memory (delayed verbal memory)	- free reproduction of the 10 terms from the word list memory test<br>- - maximum 10 points
Word list Recognition recall	Verbal memory (delayed verbal memory, recognition)	- - the 10 already mentioned terms and 10 new terms are presented to the respondent<br>- - the respondent has to declare the 10 already mentioned terms as "already known" and the 10 new ones as "not known".
Constructional praxis recall	Nonverbal memory (delayed figural	- Drawing the figures from the constructive part from memory<br>- - maximum 11 points

	memory)	
Trail-Making- Test A	Psychomotor speed,<br>Executive functions	- number linking as fast as possible (from 1-25)<br>- - max. 180 s
Trail-Making-<br>Test B	Executive functions	- combine numbers and letters alternately, keeping the numerical and alphabetical sequence, respectively (1-13; A-L)<br>- max. 300 s
Quality criteria:<br>- Reliability: Interrater reliability: ICC = 0.92 - 1.00 (American version)<br>- Test-restest reliability: good according to authors<br>-<br>- Validity:<br>o Criterion validity: for American version moderate to high correlation with other dementia measures (Ex. R = -.83 Clinical Dementia Rating Scale).<br>o Construct validity: sensitive differentiation between healthy and dementia patients and between the severity of cognitive impairment.		

Table 1

Results of the comparison between the intervention (IG) and control (CG) group

Test procedure	Group effects		
	**F (df)**	**p**	**Effect size η_p^2**
Modified chair-rising-test	F(1,55)=5.262	.026	.087
Drop-bar-test	F(1,56)=6.088	.017	.098
Hand grip strength right	F(1,57)=3.627	.062	.060
Hand grip strength left	F(1,57)=1.957	.167	.033
FICSIT-4	F(1,57)=8.702	.005	.132
Timed-Up-and-Go- test	F(1,55)=9.574	.003	.143
Verbal fluency	F(1,57)=3.216	.078	.053
Bosten-Naming-	F(1,57)=1.651	.204	.028

Test			
MMSE	F(1,57)=1.353	.250	.023
Wordlist Saving	F(1,57)=0.003	.956	.000
Discriminateability	F(1,57)=0.988	.324	.017
Constructive practice Saving	F(1,57)=3.332	.073	.055
TMT-A	F(1,57)=2.687	.108	.054
Qualidem	F(1,57)=1.423	.238	.024

Note. **FICSIT-4** Frailty and Injuries: Cooperative Studies of Intervention Techniques. **MMSE** Mini-Mental-State Examination. **TMT-A** Trail-Making-test-A. **df** degrees of freedom. **p** significance <.05.

Figure 1

Normalized values of CERAD-NP-Plus (z-values) in the intervention group at baseline (T0), three months (T1), six months (T2)

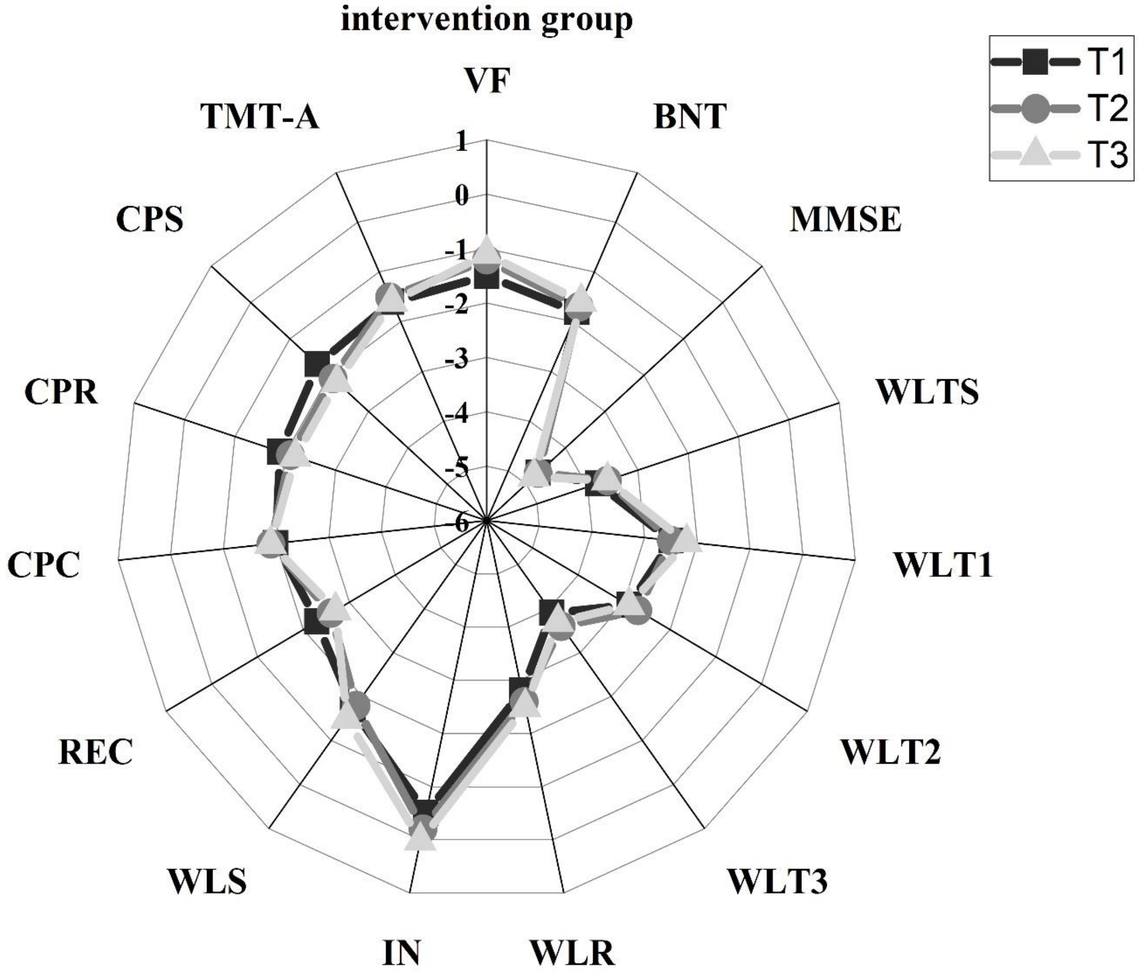

Note. **VF** verbal fluency. **BNT** Bosten-Naming-test. **MMSE** Mini-Mental-State-Examination. **WLTS** Wordlist Total. **WLT1** Wordlist Trial 1. **WLT2** Wordlist Trial 2. **WLT3** Wordlist Trial 3. **WLR** Wordlist delayed recall. **IN** Instructions. **WLS** Wordlist Saving. **REC** Recognition recall. **CPC** Constructional praxis copy. **CPR** Constructional praxis recall. **CPS** Constructional praxis Saving. **TMT-A** Trail-Making-test-A.

Figure 2

Normalized values of CERAD-NP-Plus (z-values) in the contol group at baseline (T0), three months (T1), six months (T2)

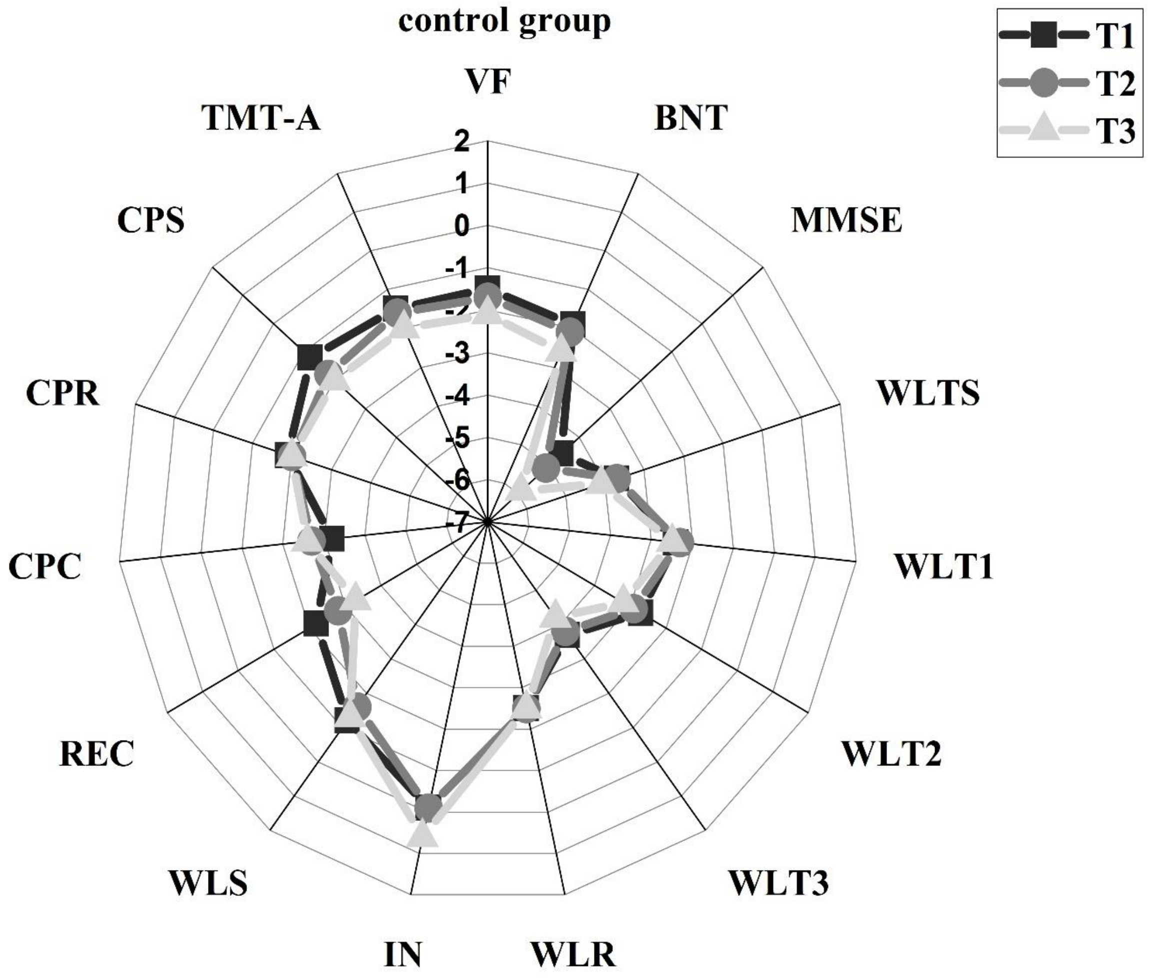

Note. **VF** verbal fluency. **BNT** Bosten-Naming-test. **MMSE** Mini-Mental-State-Examination. **WLTS** Wordlist Total. **WLT1** Wordlist Trial 1. **WLT2** Wordlist Trial 2. **WLT3** Wordlist Trial 3. **WLR** Wordlist delayed recall. **IN** Instructions. **WLS** Wordlist Saving. **REC** Recognition recall. **CPC** Constructional praxis copy. **CPR** Constructional praxis recall. **CPS** Constructional praxis Saving. **TMT-A** Trail-Making-test-A.

Table 2

motor skills test procedure

Motor function		
**Test procedure**	**Motor area**	**declaration**
modified Chair-Rising-Test<br><br>(Le Berre et al., 2016)	Strength and coordination of the leg muscles	- Measures how long it takes a respondent to stand up and sit down five times with hand assistance.<br>- Score from zero (>60s, high risk of falling) to four (<11.2s, low risk of falling)
	Quality criteria:<br>• Reliability: Interrater reliability: ICC = 0.99 - 1.00<br>o Test-retest reliability: ICC = 0.95 - 0.99<br>• Validity:<br>o Construct validity: strong convergences with physical performance (r = -0.59; Incremental Shuttle Walk Test).	
Drop bar-Test<br><br>(Fetz et al., 1978)	Elementary (simple) reactivity	- Response measured how fast the subject can grasp the falling rod (in cm)<br>- subject has three repetitions with the dominant hand<br>- best value goes into the evaluation
	Quality criteria:<br>• Objectivity: .83 - .95 (Fetz & Konexl, 1978, p.52); .72 - .91 (Richter & Beuker, 1976).<br>• Reliability: .83 - .91 (Fetz & Konexl, 1978, p.52); .58 (Richter & Beuker, 1976)	
Hand Dynamometer<br><br>(Richards et al., 1996)	Hand force<br>(current muscle force)	- grip strength is measured in both hands (in Newton)<br>- three repetitions are performed with each hand<br>- maximum value is included in the evaluation
	Quality criteria:<br>• Objectivity very high if the exact implementation rules are followed (Oltman, 2012).	

	• Reliability: .89 -.96 (Fetz & Kornexl, 1993)<br>• Content- and construct-related validity is taken as given (Oltmann, 2012)<br>• Criterion-related validity is also taken as given (Oltmann, 2012).	
Timed-Up-and-Go-Test<br><br>(D. Podsiadlo & S. Richardson, 1991)	Mobility restriction	The test person is to stand up from a sitting position without assistance, walk back and forth for 3 meters and sit down again (aids are allowed)<br><br>Score from ≤10s (Everyday mobility unrestricted) to ≥30 (Pronounced mobility restriction, usually intervention/ Need for assistive devices)
	Quality criteria:<br><br>• Reliability: Interrater reliability: ICC = 0.91 (Rydwik,2011)<br>o Test-retest reliability: ICC = 0.96 - 0.99 (Flansbierr,2005)<br>• Validity:<br>o Criterion validity: Berg Balance Scale (r=0.81), Barthel Index (r=0.78) (Podsiadlo, 1991)	
FICSIT-4<br><br>(Rossiter-Fornoff et al. 1995)	Static balance	The subject must perform four different stances with eyes open and closed (parallel, semi-tandem, tandem, and one-legged stance tests).<br><br>Each stance is performed for a maximum of 10 seconds and then scored on a 5 point scale (0 points need help to keep from falling to 4 points able to stand 10 seconds safely). At the end, all the points obtained from the stands are added up to a total score.
	Quality criteria:<br><br>• Reliability: Test-rests-reliability was good (r=.66) (Rossiter-Fornoff et al., 1995)<br>• Validity:<br>o Content validity was moderate (r=.20 - .52) (Rossiter-Fornoff et al., 1995)	

Table 3

Quality of life

**Quality of life**		
**Test procedure**	**Area**	**declaration**
Qualidem<br><br>(Ettema et al., 2007)	Quality of life	- instrument filled out by the nursing staff (external assessment)<br>- A total of 40 items distributed over the following dimensions:<br>o Care relationship<br>o Positive Affect<br>o Negative Affect<br>o Restless tense behavior<br>o Positive self-image<br>o Social relations<br>o Social isolation<br>o Feeling at home<br>o Having something to do<br>- Response options from "Very frequently" to "never<br>- high values no or only minor disturbances<br>- low values clear disturbances in the behavioral areas
Quality criteria:<br>• strong internal consistency (Cronbach's alpha > 0.7)<br>• Retest reliability: ICC > 0,7<br>• Interrater reliability: ICC > 0,7<br>• Validity:<br>o strong indications for the construct validity of the QUALIDEM<br>o Construct validity: most QUALIDEM subscales could be confirmed		
